# Surgical Management of Brain Tumors with Focused Ultrasound

**DOI:** 10.3390/curroncol30050377

**Published:** 2023-05-13

**Authors:** Yusuf Mehkri, Kevin Pierre, Samuel Joel Woodford, Caroline Grace Davidson, Ogaga Urhie, Sai Sriram, Jairo Hernandez, Chadwin Hanna, Brandon Lucke-Wold

**Affiliations:** 1 Department of Neurosurgery, College of Medicine, University of Florida, 1505 SW Archer Rd, Gainesville, FL 32608, USA; 2 Department of Radiology, College of Medicine, University of Florida, 1600 SW Archer Rd, Gainesville, FL 32608, USA

**Keywords:** focused ultrasound, brain tumors, non-invasive therapy

## Abstract

Focused ultrasound is a novel technique for the treatment of aggressive brain tumors that uses both mechanical and thermal mechanisms. This non-invasive technique can allow for both the thermal ablation of inoperable tumors and the delivery of chemotherapy and immunotherapy while minimizing the risk of infection and shortening the time to recovery. With recent advances, focused ultrasound has been increasingly effective for larger tumors without the need for a craniotomy and can be used with minimal surrounding soft tissue damage. Treatment efficacy is dependent on multiple variables, including blood–brain barrier permeability, patient anatomical features, and tumor-specific features. Currently, many clinical trials are currently underway for the treatment of non-neoplastic cranial pathologies and other non-cranial malignancies. In this article, we review the current state of surgical management of brain tumors using focused ultrasound.

## 1. Introduction

The development of focused ultrasound (FUS) was initially tailored for intracranial ablation and has recently been expanded for the treatment of various brain pathologies [1]. Ultrasounds are longitudinal (alternating compressions and rarefactions) mechanical waves that transmit their energy onto target tissues. A coupling agent, such as gel, between the transducer and biological tissue, ensures the ideal propagation of the ultrasound wave [2]. Early treatment of intracranial lesions using FUS was performed with a craniotomy since the skull caused ultrasound wave distortion, diminishing the energy transfer and efficacy [1,3]. However, the recent development of hemispheric distribution of phased arrays and concave focusing has resolved the issue of wave distortion, allowing for noninvasive procedures [3,4]. FUS acts on biological tissues through mechanical (cavitation) and thermal mechanisms of action [5]. Cavitation is the rapid formation and collapse of vapor bubbles. This leads to cellular fragmentation in a process defined as histotripsy [6]. As ultrasound travels through tissue, the wave attenuates. The diminished wave is converted to thermal energy, is absorbed by the target tissue, and ultimately increases the temperature [5]. The amount of heat absorbed can be calculated and a thermal dose can be determined. This process of sonication causes coagulation necrosis of the target tissue, subsequently ablating the targeted area [1].

Inoperable tumors can now be thermally ablated by FUS in a non-invasive manner [7]. Such minimally invasive techniques circumvent the blood loss and infection risk associated with open procedures [8]. FUS has repeatedly shown promising results in reversibly opening the BBB and allowing for the delivery of otherwise impermeable pharmaceuticals [9]. Specifically, glioblastomas are the most pernicious brain tumors. Being much more difficult to treat due to the impermeability of the BBB, FUS is being utilized to facilitate drug transport [10]. Lastly, FUS-BBB can enhance cell delivery to the brain, thereby improving the innate anticancer immune system response [11].

Regarding efficacy, the separation of the BBB is reversible and safe—with additional research fixated on drug delivery choices [12,13]. The utilization of a thermal dose to induce coagulation necrosis via ablation is promising; however, additional research needs to delineate any currently uncontrolled limitations [14,15,16]. Likewise, the cavitation process is thought to be promising; however, additional research is required to substantiate these claims [16,17]. Although FUS targeting is precise, there are limitations in targeting tumors located in the skull base or the posterior fossa [18].

In this review, we highlight the current concepts regarding the use of FUS technology for the treatment of brain tumors with an in-depth discussion on treatment indications, recent advancements, treatment optimization, and areas of ongoing investigation. This was accomplished by performing a systematic review of the literature in February 2023 using the PubMed database in accordance with Preferred Reporting Items for Systematic Reviews and Meta-Analyses. We reviewed all full-text, English articles that discussed FUS use in the surgical management of brain tumors. Using the following search terms, focused ultrasound, brain, cranial, tumor, and appropriate Boolean operators, we identified 927 articles, of which 81 met the criteria and are discussed in this article.

## 2. Indications for Use of FUS

One of the greatest obstacles to the treatment of brain tumors is the impermeability of the brain to therapeutic agents secondary to the BBB. There is a growing interest in the use of FUS to provide a non-invasive, spatially targeted method to locally increase the vascular permeability of the BBB, thus allowing for drug entry [12,19,20]. This technology is frequently combined with ultrasound contrast agents known as microbubbles in clinical studies [20,21]. When microbubbles interact with FUS sonication, microbubbles will expand and contract rapidly. This contraction results in a force generation onto the capillary walls. Gradually, the endothelial cells of the BBB will become more permeable, thereby briefly opening the BBB [19].

Rodent research [20] indicates that this technology is useful for the successful delivery of immunotherapy agents. For example, when treated with FUS, the delivery of small chemotherapy drugs in the brain tumor microenvironment was 3.9-fold higher than the control. Schoen et al. [20] also illustrated a 2.7-fold increase in antibody delivery to brain tumor models with FUS. Wei et al. [22] successfully showed an increased delivery and efficacy of the glioblastoma treatment, etoposide, alongside the opening of the BBB mediated by FUS application in rodent models. A study demonstrated the successful conversion of the immunosuppressive tumor environment to an immuno-stimulatory tumor environment in glioblastoma patients with FUS-dependent drug delivery in the presence of microbubbles [21].

Another use for FUS is to enhance liquid biopsy by enriching circulating brain-derived biomarkers. Since the BBB limits the amount of material present within the circulation, FUS-BBB opening increases cancer-related biomarkers within the circulation [23]. Meng et al. [23] found that FUS treatment enhanced extracellular vesicles of neurons and brain-specific proteins without any adverse effects. Besides the application to tumor treatment and drug delivery, FUS technology shows potential use in vessel occlusion, movement disorders, and psychiatric disorders [17].

## 3. FUS—From Past to Present—Preclinical Investigations

Ultrasound was first discovered in 1935 by Johannes Gruetzmacher by attaching a concave lens to an ultrasonic generator. In 1942, John Lynn et al. used a container with a crystal at the bottom, in which the ultrasonic wave was transmitted via transformer oil to a cellophane wrap against which the animal’s head was placed. They had to use maximal intensities to produce any cerebral effects, as noted by transient behavioral changes. However, with their treatments of 835 kHz for 5–15 min, all animals experienced significant scalp damage. They noted that a minimum time threshold at this intensity was necessary for these changes but proposed that a lower frequency would result in less superficial damage. Notably, they suggested using “a mosaic of 4 to 6 two-inch curved crystals, all focusing on a common point” an arrangement we know today as a transducer array [24].

In the early 1950s, William and Francis Fry developed a FUS device that consisted of four independent transducers with planoconcave lenses focused by a polystyrene lens to a converging focal point. The beam was transmitted to the tissue via degassed saline to avoid distortion by steaming gas bubbles. These transducers could be moved vertically to change the focal point while the animal was held in a stereotactic frame. However, the entire apparatus took up two rooms, with the controls in an upper room and the transducer array coming through the ceiling of the lower room. In their animal experiments, they made a skull flap to obviate the effects of delivering the beam through the skull. They targeted the thalamus and internal capsule in cats and showed that white matter tracts were more susceptible to the effects of FUS than grey matter [25].

## 4. FUS: Past to Present

In the 1950s, Peter Lindstrom worked with a stereotactic frame designed by Lars Leksell to study FUS as an alternative to lobotomy in patients [26]. The Fry brothers later worked with Russell Meyers from the University of Iowa to perform the first ablations in humans, targeting the substantia nigra and ansa lenticularis in Parkinson’s patients [27,28]. Even with relative successes, Lindstrom and Meyers saw that the need for a craniotomy was a limiting factor.

Lynn et al. [24] noted that the heating of the scalp limited the energy that could be applied during FUS. Clement et al. [29] solved this problem by placing transducers along a hemispherical surface to maximize the scalp surface available for absorption and reduce heating. The next development in FUS technology solved the problem of having to transmit ultrasonic waves through different tissues, which had been shown to result in phase distortions. After a 16-element needle hydrophone proved useful in monitoring acoustic feedback during ultrasonic applications [30], Hynenen and Jolesz [31] used this to calculate the distortion caused by an intact human skull at different frequencies. They found that the skull drastically weakened focal points when transducers at >1 MHz were used. However, using two transducer arrays with 64 elements, they showed that higher frequencies (up to 1.58 MHz) could still result in a sharp focal point when the phase shifts of the skull compensated for each element. This was the first time a multidimensional array was used with phase correction circuitry. Clement and Hynenen later showed that measurements from CT scans of the head could be used to calculate the phase shift of each transducer in a 320-element array, causing beams to converge within 2 mm of the intended focal point [32].

The desire to measure intracranial temperatures was paramount due to the desire to avoid unnecessary damage to surrounding brain tissue. A trial by Guthkelch et al. in 1991 [33] using flexible thermocouples during FUS therapy in patients with brain tumors showed that desired intratumoral temperatures could be achieved when a craniotomy was performed. In 1997, Hynenen et al. [34] showed that the temperature increase induced by sonications could be detected by MR thermometry. With the knowledge that a temperature of 55–60 °C was needed to cause cellular death by thermal coagulation, this lab then went on to design a model that allowed for the calculation of the lesion size induced by sequential sonications [35]. Hynenen et al. [36] brought these advances together by showing that the hemispherical transducer array could be placed in an MRI scanner to accurately measure the temperature change immediately after the sonications.

The first neurosurgical clinical trial using MRgFUS was a Phase 1 study done by Ram et al. [37] to treat patients with recurrent glioma. However, the investigators performed a craniotomy in their approach. The first transcranial application of MRgFUS occurred in 2010 when McDannold et al. [38] used the ExAblate 3000 and techniques developed by the Hynenen lab to assess the clinical feasibility of using FUS in patients with glioblastoma (GBM) [38]. They could not achieve the temperatures required for thermal ablation due to the maximum power setting of the machine at the time. Thermal ablation would have required a 1200 W sonication delivered for 20 s—longer durations had been shown to be proportional to the extent of surrounding tissue damage. In 2014, Coluccia et al. [39] reported they were able to use the ExAblate to induce thermal ablation in a patient with recurrent GBM (rGBM) by using 25 sonications of 150–950 W for 10–25 s. It has been shown that therapeutic temperatures may not be achieved in every patient treated with the same acoustic energy dose [40].

Since then, InsighTec, the first company to design a commercial MRgFUS device, has increased the number of transducer elements, increased the maximum power available, and reduced the ultrasound frequency in the ExAblate. The current version, ExAblate 4000, was FDA approved in 2021, and is currently reimbursed by insurance companies for treatment of essential tremor and Parkinson’s disease tremor. It has 1024 elements in a hemispheric transducer array encircling the head that can be electronically controlled in four degrees of movement. A detachable accessory machine pumps degassed water through this “helmet” to achieve both scalp cooling and high-fidelity transmission of the waves. The machine operates at 620–720 kHz, is compatible with multiple General Electric and Siemens MRI scanners, has MR thermometry software, and allows pre-treatment CT scans to be fused with the intraoperative MRI scan. It can achieve a maximum temperature of 58.5 °C ± 2.5 °C, with a precision of <2 mm for lesions approximately 4 mm in diameterb [2].

Another mechanism by which FUS can be used to treat brain tumors lies in opening the BBB to allow increased delivery of chemotherapeutic drugs to the tumor. The cavitation, in which microbubbles within the bloodstream are excited by low-power, pulsed sonications at the area of interest, causes shear forces in the adjacent endothelial cells that allow for the transient opening of the BBB. Hynynen et al. showed that the BBB opening induced by an ultrasound contrast agent with albumin-coated microbubbles could be identified by contrast enhancement on MRI scans [41,42]. Multiple studies have shown that various large molecules can be delivered across the BBB in this manner [43,44,45]. There are currently multiple clinical trials assessing the efficacy of using the MRgFUS-mediated BBB opening to deliver various chemotherapeutic agents [15]. An alternative to microbubbles is nanodroplets which are generated by microbubble condensation [46]. Chen et al. [47] showed that nanodroplets had a higher threshold to undergo linear oscillation (the shearing forces that open the BBB) while not showing the fragmentation associated with microbubbles at higher acoustic pressures. Histologic imaging of the brain tissue of mice in their study showed that this fragmentation caused minor cellular damage. There are currently multiple commercial microbubble manufacturers in the US.

Several companies are making devices to expand on the potential of MRgFUS, as displayed in Table 1. Work by Canney et al. [48] had shown that a catheter with multiple transducers could potentially be used to treat tumors greater than 3 cm. Acoustic MedSystem, Inc designs interstitial FUS applicators which contain multiple MRI-compatible transducers spaced evenly along their length. These transducers can be independently activated and allow for circumferential US application. The placement of the transducers allows the surgeon to configure the shape of the lesion, especially helpful with large tumors. The applicator is encased in a Celcon catheter, which allows degassed water to circulate over the transducers. McDannold et al. [49] paired this device with an automated robotic system similar to a stereotactic frame and demonstrated that the delivery of the catheters could be made more efficient.

The Sonocloud 9, designed by Carthera (Paris, France), is a miniature device with nine ultrasound transducers that is implanted in a skull window below the skin. This MRI-compatible device is attached to an external control module via a transdermal needle and allows for multiple treatments. This is currently being used in a Phase 2 trial in coordination with Northwestern University [50] to investigate the enhanced drug delivery of paclitaxel in patients with rGBM.

NaviFUS (Chang Gung, Taiwan) developed a FUS system with incorporated neuronavigation that requires a pre-treatment CT but no intra-operative MRI. Once the patient’s cranial anatomy is registered to provide a 3D anatomical image in a manner similar to Brainlab, a headpiece is placed on the patient’s head which can deliver FUS to multiple deep locations. The pre-treatment imaging allows the device to calculate the energy needed for transcranial penetration at different points. This device, designed for outpatient use, is used for BBB opening and neuromodulation, with efficacy studies underway at Chang Gung Memorial Hospital in Taiwan to enhance the delivery of bevacizumab [51] and assess the effect of combining FUS with irradiation in patients with rGBM [52]. The device allows the focal point to be adjusted up to 20 mm from a central point and incorporates a custom-designed, multi-channel, hemispherical phased array to generate beams capable of covering a large tumor volume. The location of the beams is determined using pre-treatment planning software that analyzes the patient’s CT/MRI images. The guidance of FUS energy to the desired treatment area is done with the neuronavigation tracking system, allowing for more precise treatment. This system boasts real-time control of energy output as well as feedback detection of the extent of cavitation, allowing the operator to rapidly adjust for changes in treatment.

## 5. Optimizing Treatment Efficacy

**Figure 1:**
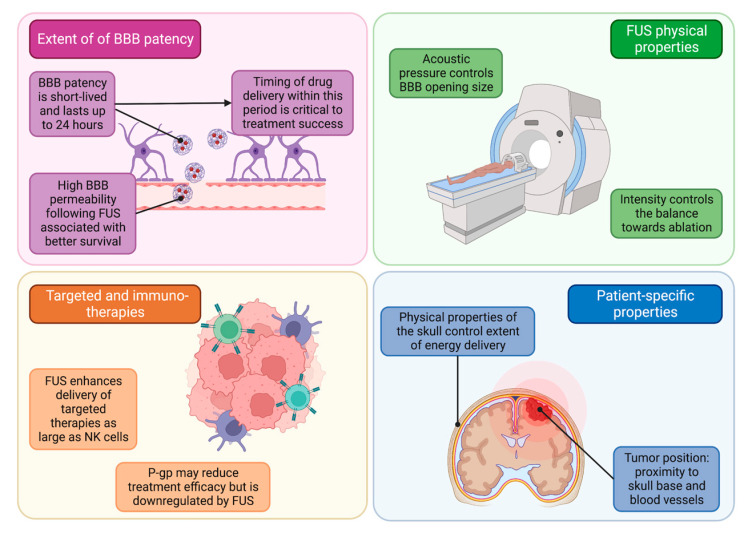
Considerations related to treatment success and failure in the use of FUS.

The use of FUS for the treatment of brain tumors is a promising non-invasive treatment modality. (Figure 1) However, optimizing the treatment of patients with FUS depends on several factors which determine the balance between treatment success and failure. In this section, we discuss some of the critical features that play a role in pharmaceutical, technological, and clinical considerations when using FUS for brain tumors.

### 5.1. Blood–Brain Barrier Patency for Pharmaceutical Delivery

One of the key features of FUS which has led to burgeoning research interest in the domain is its ability to enhance BBB permeability focally. This allows for targeted therapy entry into the tumor and peritumoral areas which would not otherwise be able to cross the BBB [53]. Importantly, the timing of chemotherapy administration is paramount to treatment success, as the focal increase in BBB permeability is transient and diminishes 20–24 h following FUS [21,53]. This, in combination with rapid drug clearance from the brain [54] underscores the importance of careful timing of therapy administration into the short-lived patent BBB period. Adaptations to FUS which increase the degree of BBB patency may enhance the delivery of antineoplastic agents and, subsequently, survival. Supporting this idea is a study of 21 patients with glioblastoma implanted with a low-intensity ultrasound device which delivered sonication monthly, immediately before the administration of carboplatin chemotherapy. Patients with radiographic evidence of low BBB patency after sonication demonstrated a greater-than-4-month reduction in the median overall survival when compared to patients with high BBB patency [55].

An additional factor associated with treatment success is the specific therapy used in conjunction with FUS. HER2-positive cancers, for example, are particularly responsive to targeted therapy such as trastuzumab [56]. MRI-guided FUS followed by systemic treatment has been recently shown to improve the delivery of this trastuzumab to brain metastases [57]. In a rat model of HER2-positive breast cancer, FUS combined with HER2-targeted NK cells improved the survival of rodents, reiterating the promising potential of FUS to deliver even large therapeutics [58]. FUS was also shown to enhance the penetration of immune checkpoint inhibitors such as antibodies to PD-L1 [59], a receptor that can be found on melanoma metastases. Of course, the non-invasive appeal of FUS may be nullified in some instances by the need to perform a brain biopsy to determine the tumor’s responsiveness to targeted agents. However, an invasive biopsy may also be circumvented in the future with the use of FUS in non-invasive blood-based brain tumor biopsy [60], though further work is required in this realm. Importantly, p-glycoprotein (P-gp) is an efflux pump which is upregulated in certain tumors, allowing the evasion of chemotherapy-induced cell death [61]. Fortunately, however, FUS has been associated with reduced expression of P-gp 24 h after sonication [62,63]. Together, these findings convey the important considerations related to the BBB in FUS.

### 5.2. FUS Properties and Patient-Specific Considerations

Several physical properties of FUS influence its efficacy. The acoustic pressure is thought to control the extent to which the BBB opens. Specifically, larger acoustic pressures induce up to 2000 kDa openings in the BBB [64]. These results suggest that acoustic pressure can be modulated to selectively allow certain pharmacologic agents to enter the peritumoral environment. The intensity of the ultrasound is another parameter that must be carefully controlled to ensure treatment effect. On one hand, low-intensity ultrasound may be used to alter BBB permeability [10], while high-intensity FUS has ablative characteristics [38].

Several anatomical and tumor-specific features are also important to consider when evaluating treatment efficacy. In one retrospective meta-analysis evaluating 25 patients who received FUS for movement disorders, increased skull volume correlated with reduced maximal temperature generated in thermal ablation. In addition, the ratio of marrow to cortical bone density was positively correlated with the temperature of thermal ablation [65]. Qualities of the tumor are also important contributors to treatment success or failure. Tumor position is one important factor since, for instance, posterior fossa or skull base tumors are particularly susceptible to bone heating [66,67]. Proximity to nerves or vessels is another important consideration as the cavitation induced by sonication can cause intracranial hemorrhage [68,69]. Taken together, the treatment success of FUS is a function of the extent of BBB permeability induced, treatment utilized, physical properties used in the FUS session, and patient-specific anatomic features.

FUS has been used in a wide variety of clinical applications including non-invasive tissue ablation, neuromodulation, and physical therapy because of its precision and ability to alter targeted tissues thermally and mechanically [2]. Developments in the clinical applications of FUS have been mediated by advancements in imaging technology that have improved the accuracy and safety of FUS treatment [70]. The imaging modalities used in conjunction with FUS are magnetic resonance imaging (MRI) and ultrasound imaging. Ultrasound-guided focused ultrasound (USgFUS) was developed in the 1970’s and continued to be the only FUS imaging method used clinically until the development of MRI-guided focused ultrasound (MRgFUS) in the mid to late 1990s [71,72]. USgFUS originally offered B-mode visualization of echogenic changes in target tissues during and immediately after FUS procedures. These low-quality images, however, lacked spatial sensitivity and could not be used for lesion localization [73].

With the implementation of MRgFUS, the soft-tissue imaging capability of MRI could be used for enhanced lesion visualization. MRI guidance also allows for real-time temperature monitoring through MR thermometry and post-procedure damage assessment through MR elastography [73,74]. The application of this technology has led to the development of FUS systems that have been approved by the FDA for the treatments of uterine fibroids, prostate cancer, osteoid osteomas, essential tremor, and tremor-dominant Parkinson’s disease. In other countries, such as China and the UK, FUS is also indicated for a variety of other solid tumor pathologies of the liver, breast, kidney, and pancreas [17]. Clinical studies are currently ongoing in the United States with the aim of expanding the clinical indications of FUS for targeted therapy. Table 2 lists all the clinical studies in the United States that are currently recruiting patients to prove the safety and efficacy of FUS (used as monotherapy or combination therapy) as a treatment for a variety of clinical conditions.

FUS has been used successfully in a wide variety of tissues [17,49,82]. It is plausible that any diagnostic ultrasound acoustic window used to visualize a target tissue can also be used for FUS treatment [83]. As such, the application of focused ultrasound is both aided by and restricted to the potential location and placement of the transducer [82]. There are currently three types of transducers being used and developed for the FUS treatment, to allow access to different target tissues and organs [17]. In most cases, when the target is accessible through an acoustic window on the skin, an extracorporeal transducer is used [84,85]. Transrectal transducers have been utilized for the treatment of prostate cancer [86,87]. Interstitial transducers are currently being investigated for potential brain tumor and esophageal cancer applications [49,88,89].

### 5.3. FUS Delivery of Immuno-Therapeutics

FUS-enhanced immuno-therapeutic delivery is an antitumor treatment that utilizes endogenous substances to eradicate cancers. These endogenous substances are immune-effector cells, such as monoclonal antibodies [11]. When considering the specific substances used, lymphokine-activated killer (LAK) cells and interleukin-2 (IL-2) displayed promising results in tumor cessation [90]. Patient survival was particularly impacted in patients suffering from glioblastomas [91]. Furthermore, interleukin-12 (IL-12) has shown great promise in enhancing th1-type cytotoxic antitumor immune responses. Although utilizing various immuno-therapeutic molecules displays effective results, the vast majority are limited by toxic side effects [92].

### 5.4. Sonodynamic Therapy

Sonodynamic therapy involves the sensitization of cancer tissues via chemical sonosensitizer agents and subsequent exposure with ultrasound [93]. Mechanistically, sonosensitizers are thought to generate radicals that initiate lipid peroxidation. The destruction of lipids destabilizes the cell membrane, rendering it increasingly vulnerable to ultrasound [94]. Although sonodynamic therapy is a recent development, pre-clinical trials indicate that sonodynamic therapy is effective in treating animal gliomas. Parameters are currently being defined and results are pending for clinical trials investigating sonodynmic therapy [95].

### 5.5. Histotripsy

Histotrispy refers to the breakdown of soft tissue through exposure to rapdily released ultrasound bursts [96]. These ultrasound bursts will cause endogenous gases to cavitate—expanding and contracting—and erode surrounding tissue [97]. To ensure the treatment is conducted specifically, ultrasound imaging can reliably guide the process [98]. In pre-clinical trials, histotripsy displayed non-invasive, specific, and positive outcomes when ablating canine tumors [99]. Within clinical studies, histotripsy has shown promise in removing prostate lesions. However, power production, beam steering, and acoustic beam resolution need to be properly modulated to further improve outcomes [100].

### 5.6. Liquid Biopsy

FUS can reversibly open the BBB and increase the amount of tumor-specific biomarkers present in peripheral circulation [101]. Therefore, a “liquid biopsy” can be obtained by drawing and analyzing plasma from the peripheral sample. This can help in the early detection of tumors, recurrence surveillance, and response monitoring. Generally, FUS uses to enhance biomarker samples have been successful [23,60,102].

### 5.7. Tumor Types

Glioblastomas are among the most aggressive brain tumors and are associated with poor outcomes [10]. FUS has been shown to enhance therapeutic treatments by attenuating the BBB. The addition of FUS has increased the overall survival of patients with glioblastomas [22]. Additionally, diffuse intrinsic pontine gliomas are surgically unresectable and demonstrate extremely low survivability—mainly impacting pediatric patients. A study investigating a rodent model demonstrated that MRgFUS increased local drug delivery [103]. Similarly, MRgFUS increased delivery in mouse models for Her2-positive brain metastases in animal models and led to decreased tumor sizes in humans [57].

## 6. Conclusions

The BBB reduces the efficacy of treatments for intracranial diseases. FUS is a powerful technique that shows the potential to transiently obviate the impermeable nature of the BBB. Furthermore, the mechanism of FUS is non-invasive and accurate. The mechanism of FUS can be modulated for varying effects. It can be used to increase the delivery of immuno-therapies, such as sonodynamic therapy, for histotripsy and to enhance liquid biopsies. Generally, FUS has increased the overall survivability for patients utilizing existing treatment. However, future studies should aim to further examine its efficacy and examine the toxicity associated with immuno-therapeutics coupled with FUS.

## Figures and Tables

**Table 1:** Logistic hurdles and solutions for MRgFUS.

Logistic Hurdles in the Development of MRgFUS	Solutions
Insufficient power at the target	Using multiple transducers in an array
Significant scalp damage	Using lower transducer frequencies
	Hemispheric transducer array Running degassed water through headpiece
Ensuring high-fidelity transmission from US transducer to tissue	Degassed saline/water
Need for craniotomy	Phased correction circuitry
Surrounding tissue damage	MR thermometry
Inability to achieve thermal ablation	Increasing maximum power output of ExAblate
Large tumors	Interstitial transducer applicators
Inability to undergo intraoperative MRI	Neuronavigation

**Table 2:** United States clinical studies using HIFU as an intervention (currently recruiting).

Clinical Study	Conditions Being Treated	HIFU Interventions
A Pilot Study: Focused Ultrasound Thalamotomy for the Prevention of Secondary Generalization in Focal Onset Epilepsy [75]	Partial Seizures with Secondary Generalization	Magnetic Resonance Guided High-Intensity Focused Ultrasound
MR-HIFU Treatment of Painful Osteoid Osteoma [76]	Osteoid Osteoma	Magnetic Resonance Guided High-Intensity Focused Ultrasound
Magnetic Resonance Guided High-Intensity Focused Ultrasound in Advanced Pancreatic Adenocarcinoma Treatment [77]	Pancreatic Adenocarcinoma	ExAblate 2100 (Magnetic Resonance Guided High-Intensity Focused Ultrasound)
Minimally Invasive Treatment of Primary Great Saphenous Vein (GSV) Insufficiency Using High-Intensity Focused Ultrasound (HIFU) [78]	Great Saphenous Vein Insufficiency	Sonovein (Ultrasound Guided Focused Ultrasound)
Treatment of Breast Fibroadenoma Targeted Tissue with HIFU [79]	Breast Fibroadenoma	ECHOPULSE (Ultrasound Guided Focused Ultrasound)
Medical Imaging and Thermal Treatment for Breast Tumors Using Harmonic Motion Imaging (HMI) [80]	Fibroadenoma Breast Cancer Stage I	Harmonic Motion Imaging Guided Focused Ultrasound
A Phase I Study of Lyso-thermosensitive Liposomal Doxorubicin and MR-HIFU for Pediatric Refractory Solid Tumors [81]	Pediatric Cancer Solid Tumors Rhabdomyosarcoma Ewing Sarcoma Soft Tissue Sarcomas Osteosarcoma Neuroblastoma Wilms Tumor Hepatic Tumor Germ Cell Tumors	Magnetic Resonance Guided High-Intensity Focused Ultrasound Lyso-thermosensitive liposomal doxorubicin
